# Features of Biochemical and Hematological Parameters and Chromosomal Disorders in Lymphocytes of Aging Primates of the Kurchatovsky Complex of Medical Primatology

**DOI:** 10.1134/S1607672925601751

**Published:** 2026-02-04

**Authors:** O. P. Chzhu, N. S. Rudenko, D. E. Araviaashvili, R. V. Panfilov, D. A. Dushin, I. I. Marinich, A. V. Popov

**Affiliations:** 1 Kurchatov Center of Medical Primatology, National Research Center Kurchatov Institute, 354376 Sochi, Russia; 2 Shemyakin and Ovchinnikov Institute of Bioorganic Chemistry, 117997 Moscow, Russia

**Keywords:** aging primates, males, females, oxidative stress, biochemical parameters, state of the antioxidant system, cellular-hematological parameters, genomic parameters

## Abstract

Aging is a key challenge for modern society. In particular, brain aging is accompanied by chronic inflammation, depletion of energy potential, and an increased level of oxidative stress, with changes in blood composition playing a special role in this process. Recent studies also show that aging progresses non-linearly throughout life. Primates are genetically and anthropometrically the closest laboratory animals to humans, thus representing the most accurate model for research. This study establishes baseline values for biochemical parameters (including the state of the body’s antioxidant system), cellular-hematological, and genomic indicators in aging primates of various species, sexes, and ages from the Kurchatov Complex of Medical Primatology. In aging males, the concentration of lipid peroxidation products was lower than in females of the same age and species. Analysis of antioxidant defense parameters indicates a more stable redox balance in old cynomolgus macaques of both sexes, which may be associated with their lower aggressiveness and high adaptability. The biochemical profile analysis in aging rhesus macaques revealed that females exhibit elevated levels of all measured parameters. In aging cynomolgus macaques, there are fewer sex-related differences in blood composition characteristics compared to rhesus macaques. It can be noted that under the housing conditions of the primates at the nursery of the Kurchatov Complex of Medical Primatology, several types of aging based on blood parameters can be observed within the same age category across different species and sexes.

## INTRODUCTION

Similar to the aging of other living beings, human aging is a biologically determined process of gradual deterioration of organ and system functions, along with the resulting consequences. The mechanisms of aging are physiologically similar to those in other mammals; however, certain aspects, particularly cognitive decline, acquire special importance in the human context. Primarily, researchers strive for a clear definition of key concepts such as aging, the period of active longevity, aging biomarkers, biological and chronological age, and the rate of aging. For instance, the period of active longevity is defined as the span of life until the onset of the first chronic disease associated with age-related changes; biological age reflects the degree of pronounced age-related biological changes; and the rate of aging represents the difference between biological and chronological age [1].

For the convenience of study, biomarkers associated with aging are typically divided into several types: molecular, biological, functional, clinical, and phenotypic. Blood can be considered an almost universal biomarker of aging, as the analysis of its components can provide insight into biological age, which reflects the actual rate of aging of the organism, unlike chronological age. The use of blood components to determine biological age is relevant in preventive medicine and gerontology, representing an effective tool for assessing age-related changes and predicting the risk of disease development. The use of these biomarkers allows for the development of individualized approaches to prevention and treatment aimed at slowing the aging process and improving the quality of life in old age [2].

Although aging itself is not classified as a disease, medications are being developed to combat age-related ailments. These ailments are characterized by increased prevalence in old age and often become the cause of death in older people. Such diseases include various types of cancer, neurodegenerative disorders such as Alzheimer’s disease and Parkinson’s disease, heart and vascular diseases, atherosclerosis, and type 2 diabetes.

Most often, aging is studied using several organisms as models: fruit flies, nematodes, or rodents. These species age relatively quickly, making them economical for laboratory use and convenient for short-term studies. Differences in maximum lifespan between various organisms in nature far exceed any lifespan extensions recorded in laboratory settings. Achievements in the laboratory, such as a 20–30% increase in the lifespan of model organisms, are significantly inferior to the natural difference in longevity observed between different biological species [3].

Molecular biologists have studied the genetic regulation of cerebral cortex development in humans and related primates from birth to death. Conducted research shows that no significant divergences in the mechanisms of genetic control between primates and humans were observed during evolution, indicating a common fundamental gene regulatory system. This means that the basic principles of how genes are regulated and function were similar in common ancestors and remain so in modern primates and humans, despite other evolutionary changes [2–4]. However, for example, lipids, which are crucial components of the brain, change significantly throughout life, with over 60% of these changes occurring before maturity. Yet, human-specific lipidomic differences persist throughout most of life and peak between the ages of 20 and 35 compared to chimpanzee-specific ones [7–8].

A group of researchers found that the rate of aging in humans and other primates remains approximately the same [9]. Recent studies indicate that the aging process is non-linear [10]. Furthermore, age groups in humans and primates coincide in concepts such as “childhood,” “youth,” “adulthood,” and “old age,” as these periods reflect similar stages of physiological development and maturation. Scientists have noted that species with longer lifespans demonstrate more synchronous mortality as they approach the limit of their life cycle. According to researchers, slowing this process is only possible by influencing the human aging rate, which has turned out to be the most stable mortality parameter [11–13].

However, in reality, the “longevity effect” is still present—a complex of factors explaining why some individuals live to a very old age. Long-livers are distinguished by the preservation of cognitive and physical functions for longer, making them a “biological elite.” Of particular interest is identifying such factors to further search for potential mechanisms of their emergence and maintenance [15, 16].

Aging is a complex process associated with almost all diseases. Understanding the molecular changes underlying aging and identifying therapeutic targets for treating age-related diseases are crucial for increasing lifespan. Many studies examine linear changes in the aging process and the prevalence of age-related diseases. However, mortality risk increases after certain time periods, indicating the importance of studying non-linear molecular changes [10].

Currently, there is no comprehensive description and, consequently, an adequate model of aging in lower primates. This hinders the use of this model for testing medications aimed at preventing age-related diseases in humans.

The Kurchatov Complex of Medical Primatology houses groups of lower primates in old age (20–30 years, corresponding to 70–100 years in humans). This provides a unique opportunity to study aging processes and develop a corresponding model. To date, information on aging processes in animals that have reached an age comparable to human age over 60 years remains fragmented and scarce.

The aim of this work was to determine and conduct a comparative analysis of the baseline values of biochemical parameters (including the state of the body’s antioxidant system), cellular-hematological, and genomic indicators in aging primates of various species, sexes, and ages from the Kurchatov Complex of Medical Primatology.

## MATERIALS AND METHODS

Biological material (whole blood, serum, plasma, hemolysate) from rhesus macaques and cynomolgus macaques of both sexes over 20 years of age, and male olive baboons over 20 years of age served as the research material.

Hematological analysis was performed on a HumaCount 30TS analyzer (Germany). Biochemical analysis was carried out on an automatic biochemical analyzer BioLit 8020 using kits from High Technology, Inc. (Business Technologies LLC, Russia).

Measurement of malondialdehyde (MDA) in blood serum was conducted by high-performance liquid chromatography using a “1100 Series” liquid chromatograph (Agilent, USA) with a diode array detector. MDA determination was performed in a gradient elution mode on a “Zorbax SB C18” column at a column temperature of 30°C, flow rate of 0.5 ml/min, and sample volume of 20 µl. Analyte detection involved derivatization using thiobarbituric acid (TBA). The reaction product is a trimethine complex with a characteristic pink color at λ_max_ = 532 nm [17].

Determination of superoxide dismutase (SOD) activity was performed in erythrocyte hemolysate by the method of inhibiting the auto-oxidation reaction of adrenaline in an alkaline medium in the presence of SOD, due to the dismutation of superoxide anion radicals, which are a product of one of the oxidation stages and simultaneously a participant in its subsequent stages [18]. The change in optical density was recorded in a cuvette with an optical path length of 10 mm (Aptaca S.p.A., Italy) at a wavelength of 347 nm every 30 s for 3 min.

Determination of catalase activity was performed in erythrocyte hemolysate by the reaction of forming a yellow-colored complex between hydrogen peroxide not destroyed during the catalase reaction and ammonium molybdate [19].

Serum protein fractions were determined by a biophysical method (acoustic analyzer AKBA-01-“BIOM,” Russia). Methods of biophysical acoustics were used to determine the components of protein fractions, measuring resonance frequencies with subsequent mathematical data processing.

Cell culturing was performed using a standard semi-micromethod for culturing human peripheral blood lymphocytes with minor modifications. The mitotic activity of cells was assessed on stained preparations by counting 1000 cells. The modal chromosome number was determined by analyzing 100 metaphase plates. The proportion of polyploid cells was determined in a sample of 1000 metaphase plates [20].

Statistical Analysis. Statistical analysis was performed in Statistics 10 software. Normality of distribution was checked using the Shapiro-Wilk test. Statistical significance was determined using the Mann–Whitney U-test for comparing two groups.

## RESULTS AND DISCUSSION

### Indicators of the Body’s Antioxidant System Status

Malondialdehyde (MDA) is a marker of oxidative stress, formed during lipid peroxidation. Its elevated level indicates damage to cells and tissues in the body.

Regulation of lipid peroxidation at the initiation stage is carried out by enzymes: superoxide dismutase (SOD) and ceruloplasmin (ferroxidase); at the chain branching stage: by catalase, peroxidase, glutathione peroxidases, and glutathione transferases.

Superoxide dismutase (SOD) converts the toxic superoxide anion (${\mathrm{O}}_{2}^{-}$) into more stable hydrogen peroxide (H_2_O_2_) and oxygen, and catalase then decomposes this H_2_O_2_, which can be harmful itself but is less aggressive than superoxide, into harmless water and oxygen. Thus, SOD and catalase work in tandem, forming the first and second lines of defense against reactive oxygen species, preventing oxidative stress in cells.

**Fig. 1.**
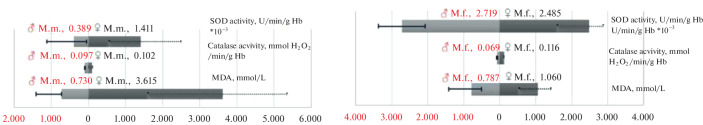
Antioxidant defense system parameters in male and female aged rhesus (M.m.) and Javan (M.f.) macaques (median by group with interquartile range (Q1–Q3)).

Oxidative stress can result from an increased production of potentially dangerous reactive oxygen species and/or a reduced ability to counteract damage through antioxidants. In this study, differences in oxidative damage between and within sexes were most likely associated with the first and second factors, as changes in lipid peroxidation indicators and antioxidant activity levels were observed (Fig. 1).

It is classically considered that females possess increased resistance to the damaging effects of oxidative stress, which is attributed to their hormonal background, particularly the presence of estrogen, which exhibits antioxidant activity.

Study [21] assessed oxidative stress in free-ranging, provisioned rhesus macaques on Cayo Santiago, a small island off the coast of Puerto Rico. The mean age of females was 9.4 ± 0.9 years (range 5.4–18.9), and of males was 8.6 ± 0.7 years (range 5.2–15.2). The noted differences in lifespan between the sexes were linked by the researchers to gender-specific costs driven by intrasexual competition and efforts directed at reproduction. Given that male rhesus macaques experience more intense intrasexual competition and have a shorter lifespan, it was hypothesized that they would experience greater oxidative stress than females, and that this stress would reflect sex-specific measures of reproductive activity.

In males, unlike females, elevated concentrations of 8-OHdG and malondialdehyde were found, which serve as indicators of oxidative DNA damage and lipid peroxidation, respectively. In females who had given birth multiple times and nursed daughters, the level of 8-OHdG was higher than in females with sons. However, no differences in antioxidant activity were detected.

Lipid peroxidation was higher in males (MDA concentration was 0.663 µmol/L) than in females (MDA concentration was 0.613 µmol/L), but was not associated with age. It should be noted that the interquartile range of values indicates the influence of individual factors among the subjects, with this range being more pronounced towards increased lipid peroxidation values in males.

**Fig. 2.**
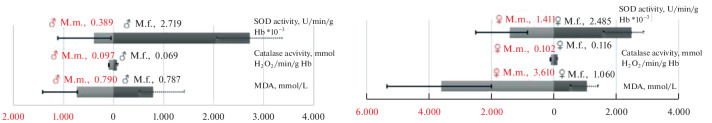
Antioxidant defense system parameters in male and female aged rhesus (M.m.) and Javan (M.f.) macaques (median by group with interquartile range (Q1–Q3)).

In the present study, which investigated primates kept in captivity and over 20 years of age, lipid peroxidation in males was lower than in females of the same species and age (MDA concentration in male rhesus macaques was 0.730 µmol/L, in females—3.615 µmol/L; MDA concentration in male cynomolgus macaques was 0.787 µmol/L, in females—1.060 µmol/L), while the interquartile range, confirming the influence of individual factors, persists (Fig. 2).

Study [22] demonstrated that women with a longer reproductive period had nearly 20 percent higher levels of 8-OHdG and 60 percent higher levels of SOD, indicating antioxidant defense, compared to women with a shorter reproductive period. Furthermore, the levels of 8-OHdG and SOD were directly proportional to the duration of reproductive life, suggesting a dose-effect relationship between reproductive activity and oxidative stress.

Increased DNA oxidation in women with more active reproductive lives indicates an imbalance between the generation of free radicals and the repair of the damage they cause, pointing to intensified oxidative stress in response to increased reproductive load. Similarly, elevated levels of SOD, a key enzyme protecting against free radical effects, also indicate enhanced generation of reactive oxygen species and should be interpreted as a marker of increased oxidative stress. The authors suggest that the cumulative effect of reproductive efforts increases susceptibility to oxidative stress in women during the postmenopausal period.

In our case, when studying the biological material of female rhesus macaques and cynomolgus macaques over 20 years of age living in captivity, comparative analysis reveals elevated levels of MDA and SOD, which likely confirms the hypothesis of a cumulative effect of reproductive efforts on oxidative stress parameters in the postmenopausal period.

Analysis of the body’s antioxidant defense system status indicators reveals a more homogeneous redox status in elderly cynomolgus macaques of both sexes (MDA concentration in males was 0.787 µmol/L, in females—1060 µmol/L; SOD activity in males was 2.719 × 10^3^ U/min/g Hb, in females—2.485 × 10^3^ U/min/g Hb).

In elderly rhesus macaques, a decrease in antioxidant activity is observed compared to cynomolgus macaques (SOD activity in males was 0.389 × 10^3^ U/min/g Hb, in females—1.411 × 10^3^ U/min/g Hb), while female rhesus macaques exhibit enhanced accumulation of peroxide forms (MDA concentration in males was 0.730 µmol/L, in females—3.610 µmol/L).

Typically, cynomolgus macaques are characterized by low aggression levels and high adaptability, and are kept in family groups. Rhesus macaques demonstrate higher aggression levels, and their behavior can be more complex, requiring more specific conditions. In groups consisting of adult males, the highest frequency of lethal aggression was noted in rhesus macaques. Males exhibited aggression and were subjected to aggressive attacks equally, whereas females were significantly more often subjected to aggression than they initiated it themselves [23, 24]. This may be linked to the significant MDA accumulation in female rhesus macaques, as well as the decreased activity of their body’s defense system parameters.

The second line of defense against reactive oxygen species, determined by catalase activity, is lower in females than in males.

**Composition of protein fractions.** Elevated values of total serum protein concentration are often detected in cases of prolonged ongoing diseases, as well as during inflammatory reactions characterized by stimulation of immune defense and an increase in immunoglobulin concentration.

Pathological changes in the levels of globulin protein fractions are an important diagnostic sign. Alpha-globulins increase during allergic reactions, inflammation, stress, and trauma.

If the level of beta-fractions in the body increases, it may indicate a disease accompanied by lipid accumulation in the body, as well as pathologies of the cardiovascular system.

Quantitative changes in gamma-globulins are of particular importance. Gamma-globulins include acquired and natural antibodies responsible for humoral immunity. Decreased levels of gamma-globulins may indicate prolonged chronic diseases with suppression of the immune system. Increased levels of gamma-globulins may point to diseases characterized by activation of the immune system.

A sharp increase in globulin content in the blood of animals occurs during infectious diseases and acute inflammatory processes, since immune bodies and antitoxins are by nature gamma- and beta-globulins and accumulate in the blood of animals during immunization.

Unlike other species of laboratory animals, cynomolgus macaques exhibit significant variation and elevated values of total protein and globulins.

Albumin concentration in cynomolgus macaques can serve as a marker of their general well-being. For instance, individuals experiencing nutritional deficiencies, developmental delays, or gastrointestinal problems often show reduced levels of this protein fraction [25]. Albumin neutralizes free radicals and toxins, such as endotoxins, helping to protect cells and tissues from damage.

In older age groups, there is an increase in the number of individuals with low albumin levels, who experience a natural decline in liver protein synthesis [26]. In the conducted study, albumin content varied within the range of 30 to 40 g/L, which is consistent with data from study [27]. Furthermore, the difference in values between male and female cynomolgus macaques was more pronounced than in rhesus macaques—female cynomolgus macaques had higher albumin content than males. The proportion of albumin to total protein is higher for females of both species.

**Fig. 3.**
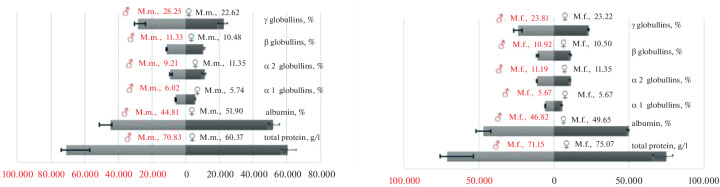
Protein fractions in male and female aged rhesus (M.m.) and Javan (M.f.) macaques (median by group with interquartile range (Q1–Q3)).

An increase in gamma-globulin levels in older age groups indicates metabolic changes occurring in the body. Oxidative damage to proteins, nucleic acids, and lipids accumulates in cells, leading to alterations in their structure and functional properties [26]. Comparative analysis of the protein fraction composition in aged animals revealed an enhanced immune system response, which may indicate the presence of inflammatory processes (Fig. 3). Such processes are known to often accompany aging and can significantly affect the overall health status of the animals. The proportion of gamma-globulins to total protein is higher in male rhesus macaques, indicating the accumulation of inflammatory processes. In cynomolgus macaques, a slight difference in gamma-globulin values is observed between males and females.

In study [27], the total albumin/globulin ratio in male cynomolgus macaques aged 18–27 years was significantly lower than in males aged 4–9 years; a decrease in this indicator in females was observed at the level of a trend. In this study, it can be noted that the total albumin/globulin ratio for male macaques of both species is lower than the corresponding value for females.

### Quantitative (Genomic) Assessment of Chromosomes

With age, not only does the frequency of chromosomal aberrations increase, but the functional activity of cells also deteriorates, which can ultimately lead to the development of various diseases; furthermore, the level of oxidative stress increases. Oxidative damage is one of the key factors contributing to aging.

All analyzed cell karyotypes showed significant stability in chromosome number, namely the modal chromosome number was predominantly diploid: 2*n* = 42.

**Fig. 4.**
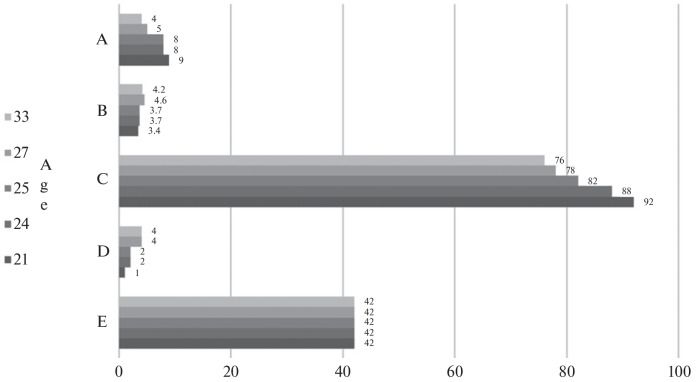
Results of the quantitative genomic study of biological material from aging primates ((a) mitotic activity (number of metaphase plates per 1000 cells); (b) proportion of polyploid cells, %; (c) proportion of cells with modal chromosome number, %; (d) range of variability in chromosome number; E—modal number of chromosomes). The age of the research object is shown in grayscale.

Histograms illustrating the distribution of cells by chromosome number were constructed based on the results of counting 100 routinely stained metaphase plates. A small range of chromosome variability (1–4 chromosomes), observed in cell cultures, was due to random chromosome losses during the karyotyping process and varied depending on age, not exceeding the modal chromosome number (Fig. 4).

The proportion of cells with polyploidization varied among the monkeys according to age, while the mitotic index was not high, which corresponds to established data on the decrease in cell growth during aging.

Furthermore, comparative analysis revealed the following trends: a decrease in mitotic activity and the proportion of cells with the modal chromosome number with age, and an increase with age in the proportion of polyploid cells and the chromosome variability interval.

The decrease in mitotic activity may be associated with the depletion of the stem cell pool, as well as the activation of cellular senescence as a protective mechanism against malignancy. The decrease in the proportion of cells with the modal chromosome number is likely a consequence of chromosomal instability, leading to the loss or acquisition of individual chromosomes during division.

The increase in the proportion of polyploid cells indicates a disruption of cytokinesis during cell division. The mechanisms controlling chromosome segregation and cytoplasmic division may become less accurate with age, leading to the formation of cells with a doubled or higher chromosome set. The chromosome variability interval, which widens with age, demonstrates increasing heterogeneity of the cell population in terms of karyotype [28].

### Biochemical and Hematological Parameters

Biochemical and hematological parameters, reflecting metabolic processes and blood status, play a key role in assessing age-related changes and identifying age-associated pathologies.

**Fig. 5.**
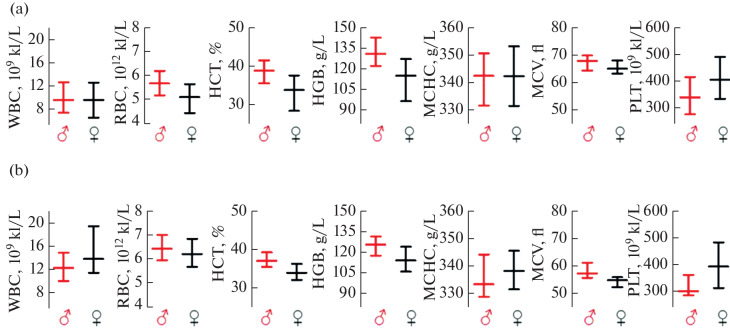
General blood test analysis for male and female age groups of M.m. (a) and M.f. (b) (median values for the group with interquartile range (Q1–Q3)).

Conducting a comparative analysis of the hematological parameters of primates of both sexes and species, the following trends can be noted (Fig. 5):

1. The hemoglobin value in female aging primates is lower than in males, while the value intervals between the lower and upper limits of the indicators do not differ significantly for both sexes respectively.

2. The platelet count in female aging primates is higher than in males, while the values of the lower and upper limits of the indicators in females of both species are close to each other.

3. The level of hemoglobin and the concentration of platelets in the blood are closely related and can reflect the overall condition of the body. A decrease in hemoglobin may mean that the body is experiencing a lack of oxygen, and an increase in platelet concentration may indicate the presence of inflammation, infection, pancreatitis in the body, and also occurs as the body’s response to stress or blood loss [27].

4. The leukocyte count in female and male aging rhesus macaques and cynomolgus macaques has close values and intervals between the lower and upper limits.

5. The erythrocyte count in aging rhesus macaques is somewhat lower than the corresponding values in cynomolgus macaques for both the lower and upper limits.

**Fig. 6.**
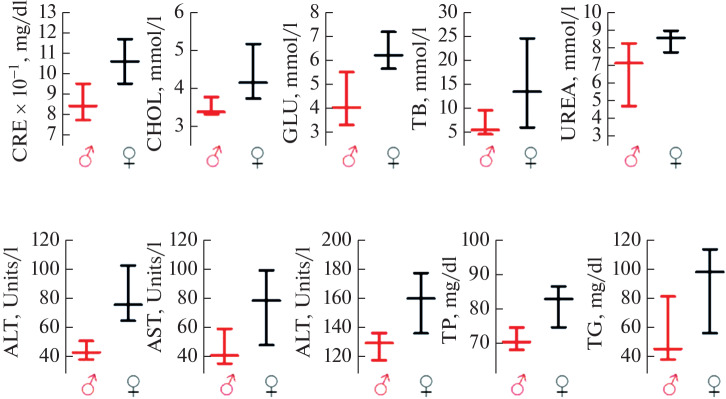
Biochemical blood analysis of male and female age-matched M.m. (median value for the group with interquartile range (Q1–Q3)).

Evaluation of biochemical parameters in aging rhesus macaques showed that all determined parameters for females have higher values (Fig. 6). The results of the biochemical analysis of the blood of aged Javanese macaques of the same group were presented earlier in the work [27]. These data indicate sexual dimorphism in biochemical parameters in aging rhesus macaques, which may be associated with age- and sex-related changes in metabolism, liver, kidney, and other organ functions, as well as habitat, diet, and psychoemotional state. It is noteworthy that in aging females compared to males, the ALT and AST values at the upper and lower limits of the value interval are 1.5–2 times higher.

A similar situation is observed for glucose and triglyceride levels. Modern research suggests that the triglyceride-glucose index may be an independent prognostic parameter for the development and progression of various cardiovascular diseases, including coronary artery disease, stroke, heart failure, and peripheral atherosclerosis. Furthermore, the reasons for a high index may include not only diabetes mellitus, atherosclerosis, acute and chronic pancreatitis, obesity, but also metabolic syndrome and severe chronic stress [30].

An elevated triglyceride-glucose index is associated with an increased risk of sudden cardiac death and all-cause mortality. In this context, it is interesting to consider the model of aging rhesus macaque primates with pronounced sexual and age-related gradation in glucose and triglyceride levels [31].

## CONCLUSIONS

A comparative analysis of the biochemical, hematological, and chromosomal composition of blood in primates over 20 years of age, of various species and sexes, living in captivity at the nursery of the Kurchatov Complex of Medical Primatology, revealed a number of the following features.

In the present study, variations in oxidative stress observed between and within sexes were most likely due to a complex of interrelated factors, as fluctuations in lipid peroxidation parameters and antioxidant enzyme activity were established.

In aging males, the concentration of lipid peroxidation products was lower than in females of the same age and species.

A comparative analysis of female rhesus and cynomolgus macaques revealed elevated concentrations of malondialdehyde (MDA) and superoxide dismutase (SOD), which may confirm the assumption of a cumulative effect of reproductive load on oxidative stress parameters in the postmenopausal period.

Analysis of the body’s antioxidant defense parameters indicates a more stable redox balance in old cynomolgus macaques of both sexes, which may be associated with their lower aggressiveness and high adaptability.

Aging rhesus macaques exhibit decreased antioxidant activity compared to cynomolgus macaques, while female rhesus macaques show increased accumulation of lipoperoxidation products. This is likely due to the fact that female rhesus macaques throughout their lives were significantly more often victims of aggression rather than initiators, leading to a significant increase in MDA content and a weakening of antioxidant system enzyme activity.

Catalase activity, which is the second line of defense against free radicals, is lower in females than in males.

Among elderly individuals, there is a trend towards an increase in the number of individuals with albumin deficiency, associated with a natural decrease in its production by the liver. In cynomolgus macaques, gender differences in albumin concentration are more pronounced than in rhesus macaques—female cynomolgus macaques have higher albumin levels than males. The relative albumin content in the total protein fraction is higher in females of both species.

An increase in gamma-globulin concentration in older age groups indicates metabolic shifts in the body. The relative content of gamma-globulins in the total protein is higher in male rhesus macaques, which is a sign of the accumulation of inflammatory phenomena. In cynomolgus macaques, the differences in gamma-globulin concentration between males and females are weakly expressed.

This study established that the albumin-to-globulin ratio in male macaques of both species is lower than in females. This fact also suggests the presence of inflammatory processes or other pathological conditions in the bodies of males.

A comparative study of chromosomes revealed that with increasing age, the intensity of cell division (mitosis) and the number of cells with a typical chromosome set decrease. At the same time, there is an increase in the proportion of cells with a multiple chromosome set (polyploid) and an expansion of the range of variations in chromosome number. The identified patterns indicate the gradual accumulation of genetic material damage and disruptions in the processes controlling cell division as the organism ages.

In adult female primates, blood hemoglobin concentration is generally lower than in males, although the range of value fluctuations (from minimum to maximum) is comparable for both sexes. At the same time, the platelet count in old females is higher than in males, and the lower and upper limits of normal values for females show similar indicators. A decreased hemoglobin level may indicate insufficient oxygen saturation of tissues. An increase in platelet count often serves as a sign of inflammatory processes in the body and can also be a reaction to stressful situations or blood loss.

Analysis of the biochemical profile in aging rhesus macaques revealed that females have elevated levels of all measured parameters. It is especially important to note that the activity of ALT and AST enzymes in old females, both in minimum and maximum values, exceeds the corresponding indicators in males by 1.5–2 times. A similar trend is observed for serum glucose and triglyceride concentrations. Current scientific works indicate that the triglyceride-glucose index can serve as an independent predictor of the occurrence and progression of a number of cardiovascular pathologies, such as coronary artery disease, stroke, heart failure, and peripheral artery atherosclerosis. An additional factor influencing the increase in this index may be chronic stress. In this regard, it seems interesting to study the age model of primates—rhesus macaques, with a focus on pronounced sexual and age-related differences in glucose and triglyceride levels.

In general, it can be noted that under the housing conditions of the primates at the nursery of the Kurchatov Complex of Medical Primatology, different models of aging can be observed in different species and sexes. Aging cynomolgus macaques are characterized by fewer differences in blood composition features between sexes compared to rhesus macaques. At the same time, the load of oxidative stress is more reflected in the parameters of females and is more pronounced in aging female rhesus macaques. It can be noted that within the same age category under these housing conditions, several types of aging based on blood parameters can be observed across different species and sexes.

The data can be used in research on the mechanisms of blood-based aging in primates, as well as in the development and testing of therapeutic agents.
